# Monocyte-to-Lymphocyte Ratio as New Prognostic Factor in Patients with Medullary Thyroid Carcinoma

**DOI:** 10.3390/jcm15062363

**Published:** 2026-03-19

**Authors:** Luca Canali, Francesca Gaino, Claudia Valenziano, Giulio Sandri, Alberto Paderno, Fabio Ferreli, Luca Malvezzi, Gherardo Mazziotti, Andrea Lania, Giuseppe Spriano, Giuseppe Mercante

**Affiliations:** 1 Department of Biomedical Sciences, Humanitas University, Via Rita Levi Montalcini 4, 20072 Pieve Emanuele, MI, Italy; 2 Otorhinolaryngology Unit, IRCCS Humanitas Research Hospital, Via Manzoni 56, 20089 Rozzano, MI, Italy; 3 Endocrinology, Diabetology and Medical Andrology Unit, IRCSS Humanitas Research Hospital, Via Manzoni 56, 20089 Rozzano, MI, Italy

**Keywords:** thyroid cancer, medullary thyroid carcinoma, prognostic factor, immune system, monocyte-to-lymphocyte ratio

## Abstract

**Objectives:** Medullary thyroid carcinoma (MTC) is a rare but biologically aggressive neuroendocrine tumor for which reliable preoperative prognostic biomarkers are still lacking. This study aimed to evaluate the association between preoperative blood immunological markers and disease recurrence in patients with MTC undergoing curative surgery. **Methods:** We conducted a retrospective cohort study at a single tertiary academic center including 52 consecutive patients who underwent curative surgery for MTC between January 1999 and December 2023. The study size was determined by including all eligible consecutive patients meeting predefined inclusion/exclusion criteria within the study period. Preoperative inflammatory indices (MLR, NLR, PLR, SII, SIRI) were calculated from standardized complete blood count tests performed within 30 days before surgery. Disease-free survival (DFS) was calculated using the Kaplan–Meier method. Cox proportional hazards regression analysis with a backward stepwise selection based on the Akaike Information Criterion was used to identify independent predictors of recurrence, adjusting for potential confounders. **Results:** The mean age was 55.0 years (range 31–75), and 73% of patients were female. The ROC-derived cut-off for preCT was 181 pg/mL. Locally advanced disease (T3-T4) was observed in 12% of cases, and cervical node metastases in 27%. With a mean follow-up of 75.48 months, the 3- and 5-year DFS rates were 91% and 86%, respectively. On multivariable Cox regression, a high monocyte-to-lymphocyte ratio (MLR ≥0.37), positive surgical margins, and pathological nodal involvement remained independently associated with worse DFS after confounder adjustment (HR 9.73, 10.78, and 17.71, respectively). **Conclusions:** Elevated MLR, histological node metastases, and positive surgical margins independently predict recurrence in MTC after curative treatment. Preoperative MLR may represent a simple, inexpensive, and reproducible biomarker to improve preoperative risk stratification and personalize surgical and follow-up strategies: patients with MLR ≥0.37 may benefit from more aggressive management and/or closer follow-up.

## 1. Introduction

Medullary thyroid carcinoma (MTC) is a rare thyroid cancer, accounting for approximately 1–2% of all thyroid nodules and 3–5% of thyroid malignancies, with an estimated incidence of 0.2–0.4 per 100,000 per year [1,2]. Unlike differentiated thyroid carcinomas, MTC does not concentrate radioiodine and therefore lacks effective adjuvant radioactive treatment options [2]. It originates from the C cells and generally secretes calcitonin (CT), a highly specific tumor marker [3]. Serum calcitonin (CT) and carcinoembryonic antigen (CEA) represent the main circulating biomarkers used for diagnosis and follow-up [2]. Preoperative CT levels correlate with tumor burden and nodal metastases; however, no universally accepted cut-off values reliably guide the extent of elective neck dissection [4,5,6]. Furthermore, doubling times of CT and CEA are recognized prognostic indicators during follow-up, but their preoperative predictive value for recurrence remains debated. Surgery is the only curative treatment, especially when the disease is confined to the thyroid or has minimal node spread. Recommended treatment includes total thyroidectomy, central neck dissection (CND), and dissection of involved lateral neck compartments (LND) [1]. The 2015 American Thyroid Association Guidelines recommend the consideration of dissection of negative neck compartments based on preoperative serum CT levels [2]. However, there is no consensus on the CT cut-off to guide surgical aggressiveness [5], nor on the benefits of elective neck dissection (END) based on preoperative CT levels [6]. Furthermore, more extensive neck dissection, particularly in the central compartment, increases risks, including bleeding, hypocalcemia, and nerve injury [7]. Therefore, the identification of other prognosticators that can select high-risk patients who could benefit from more aggressive elective treatment and more stringent follow-up is warranted.

Blood immunological markers, like the monocyte-to-lymphocyte ratio (MLR), have been strongly associated with disease aggressiveness, recurrence risk, treatment response, and survival in various malignant tumors [8]. They represent promising markers because of their cost-effectiveness, broad availability, and ease of calculation. Although inflammatory indices such as the neutrophil-to-lymphocyte ratio (NLR), platelet-to-lymphocyte ratio (PLR), and lymphocyte-to-monocyte ratio (LMR) have demonstrated prognostic value in several solid tumors, data in MTC are limited and mainly derived from small pilot studies with heterogeneous endpoints. Therefore, additional validation in well-characterized surgical cohorts with long-term follow-up is warranted.

This single-center retrospective study aims to evaluate whether blood inflammatory and immunological markers correlate with recurrence risk in patients with MTC who underwent curative treatment.

## 2. Materials and Methods

### 2.1. Study Population

A single-center retrospective cohort study was conducted at Humanitas Research Hospital (Rozzano, Italy), including consecutive MTC patients who underwent total thyroidectomy or hemithyroidectomy between January 1999 and December 2023. The study size was determined by including all consecutive patients meeting eligibility criteria during the study period. Given the rarity of MTC, no formal a priori power calculation was feasible; however, the inclusion of all eligible cases minimized selection bias and reflects real-world clinical practice in a tertiary referral center. Inclusion criteria were: (1) age ≥18 years; (2) histologically confirmed MTC; (3) surgery with curative intent (total thyroidectomy or hemithyroidectomy ± neck dissection); and (4) availability of complete preoperative blood count within 30 days before surgery. Exclusion criteria included: (1) age <18 years; (2) prior thyroid surgery performed elsewhere; (3) distant metastasis at diagnosis; and (4) biochemical, histopathological or follow-up data missing.

The study followed the Transparent Reporting of a Multivariable Prediction Model for Individual Prognosis or Diagnosis (TRIPOD) guidelines. The study respected the Helsinki Declaration and was approved by the Institutional Review Board, which waived the need for informed consent due to its retrospective nature.

### 2.2. Patient Management Protocol

All patients underwent total thyroidectomy or hemithyroidectomy. Patients received CND when there was a definite or highly suspected preoperative diagnosis of MTC. LND was reserved for patients with preoperative clinical involvement of the compartment. Preoperatively, patients underwent thyroid and neck ultrasound and comprehensive blood tests within one month prior to surgery at our center [1,2]. All surgeries were performed by surgeons specialized in thyroid pathology. The specimens were analyzed by dedicated pathologists, and histological diagnoses were made following the World Health Organization (WHO) criteria [9]. Preoperative and postoperative TNM classifications were based on the eighth edition of the American Joint Committee on Cancer (AJCC) manual [10,11]. Postoperative endocrinological evaluations, along with blood tests (mainly for CT and carcinoembryonic antigen [CEA]), were conducted at 1 and 3 months, followed by regular clinical and radiological follow-up as needed: at 1- to 3-month intervals during the first postoperative year, 3- to 6-month intervals during the second and third years, and 6- to 12-month intervals from the fourth year [1,2].

### 2.3. Data Collection

For each included patient, data regarding gender, age at the time of surgery, and comorbidities such as smoking status, diabetes mellitus (DM), and hypertension (HTN) were collected. Data from preoperative ultrasounds were extracted, including the ultrasound dimensions of the suspicious nodule, echogenicity, and the presence of microcalcifications. However, considering that these ultrasound data were not available in all reports and that this procedure is highly operator-dependent, ultrasound characteristics were not included in the formal analysis. Preoperative TNM status was collected when specifically documented in the medical record or inferable. Due to the low confidence in this data and the absence of the TNM status in many patients, these variables were not included in the formal analysis. When extracting preoperative blood test data, including preoperative calcitonin levels (preCT) and inflammatory markers, the most recent test performed before surgery was considered. The following inflammatory markers were calculated: monocyte-to-lymphocyte ratio (MLR) as the absolute monocyte count divided by the absolute lymphocyte count [8,12,13]; neutrophil-to-lymphocyte ratio (NLR) as the absolute neutrophil count divided by the absolute lymphocyte count [14,15]; platelet-to-lymphocyte ratio (PLR) as the absolute platelet count divided by the absolute lymphocyte count [15]; systemic immune inflammation index (SII) as the absolute platelet count multiplied by the absolute neutrophil count and divided by the absolute lymphocyte count [16]; and systemic inflammation response index (SIRI) as the absolute neutrophil count multiplied by the absolute monocyte count and divided by the absolute lymphocyte count [17]. From the final histological reports, the variables extracted included histological tumor size (Hdim), multifocality, angioinvasion, extrathyroidal extension (ETE), and surgical margin involvement (R status). For completeness, the number of nodes retrieved from each compartment (central or lateral) and those positive for tumor metastasis were collected, although they were not considered in the formal analysis. Additionally, pathological TNM classification was recorded. In the case of multifocal tumors, the largest lesion was considered for the variable Hdim. CT normalization was defined as blood CT levels returning to the normal range (0.0–5.5 pg/mL according to the institutional laboratory reference range) at the first postoperative control [2,4]. When available, clinically significant RET mutation status was extracted and reported in the dataset. Disease recurrence was defined as structural and/or biochemical evidence of disease, confirmed by definitive histological examination if possible [1,2]. Survival periods were calculated from the date of surgery to the date of recurrence or the latest available follow-up evaluation.

### 2.4. Statistical Analysis

The following variables were included in the survival analysis: age, sex, smoking status, DM, HTN, preCT, MLR, NLR, PLR, SII, SIRI, Hdim, ETE, angioinvasion, R status, multifocality, pathological T and N classifications, and CT normalization. Missing data for these relevant variables, which accounted for 1.19% of the dataset, were imputed using regression-based imputation models appropriate to the variable type. Cut-off values for preCT, Hdim, MLR, NLR, PLR, SII, and SIRI were determined using receiver operating characteristic (ROC) curve analysis. Categorical variables were summarized as absolute counts and percentages, while continuous variables were reported as the mean and range. Furthermore, 3- and 5-year disease-free survival (DFS) rates were estimated using the Kaplan–Meier method. The log-rank test was used to compare survival distributions between patient subgroups. Univariate analysis for DFS was performed using a Cox proportional hazards model, and hazard ratios (HRs) with 95% confidence intervals (CIs) were calculated. Variables with a *p*-value < 0.10 in univariate analysis were included in the multivariate analysis. Potential confounders considered in the multivariable model included age, sex, preCT, Hdim, ETE, angioinvasion, R status, pT status and pN status. To avoid multicollinearity, correlations between variables were assessed using Pearson’s correlation coefficient. Highly collinear variables (e.g., MLR and SIRI) were not entered simultaneously into the final model. A multivariable Cox proportional hazards model was applied using a backward stepwise selection procedure. At each step, the least significant variable was removed, and the process continued until no further improvement in the Akaike Information Criterion (AIC) was observed, or only variables with significant associations remained in the model. Statistical analyses were performed using RStudio (version 2024.09.0-375), R (version 4.4.1), and Python (version 3.12.4). A *p*-value < 0.05 was considered statistically significant.

## 3. Results

Fifty-two patients were retrospectively enrolled in the analysis. The characteristics of the cohort are detailed in Table 1.

The mean age was 55.0 years (range 31–75), and 38 patients (73.1%) were female.

In total, 49 patients (94.2%) underwent total thyroidectomy, of whom 42 (80.8%) also had CND, and 3 patients (5.8%) underwent hemithyroidectomy. In 10 patients (19.2%), there was no preoperative suspicion of MTC. Three patients underwent ipsilateral LND, while 2 underwent bilateral LND. One patient had an incidental diagnosis of MTC during a total thyroidectomy and LND for clinically evident cervical localization of papillary thyroid carcinoma. In this specific case, the neck dissection was performed only on the contralateral side relative to the localization of the MTC.

The mean preCT level was 570.90 pg/mL (range 1.40–4983.50 pg/mL).

Preoperative ultrasound characteristics of the histologically confirmed tumors were available for only 37 patients (71.2%). Details regarding ultrasound findings are shown in Table 1.

In 47 cases (90.4%) the tumor was in one of the thyroid lobes (27 on the right and 20 on the left), while in the remaining 5 cases (9.6%) the tumor was bilateral and multifocal. The mean Hdim was 12.29 mm (range 1–55 mm). ETE, angioinvasion, and positive surgical margins were detected in 4 (7.7%), 11 (21.2%), and 4 (7.7%) cases, respectively. At the pathological evaluation, 12 patients (23.1%) had node involvement. Positive nodes were found in the central compartment in 11 cases and in lateral levels in 4 cases, 2 of which were bilateral.

Postoperatively, CT levels normalization occurred in 39 of 49 evaluable cases (79.6%).

RET mutations were identified in 6 out of 39 (15.4%) patients for whom this data was available.

The mean follow-up period was 75.48 months (range 2–300 months), during which 9 recurrences occurred: 8 involved regional neck nodes, and 1 was a distant pulmonary metastasis.

The cut-off values for preCT, NLR, MLR, PLR, SII, SIRI, and Hdim calculated with ROC analysis were 181.00 pg/mL, 2.46, 0.37, 193.12, 418.7, 1.56, and 11 mm, respectively.

The overall 3-year and 5-year DFS rates were 91% and 86%, respectively.

**Figure 1:**
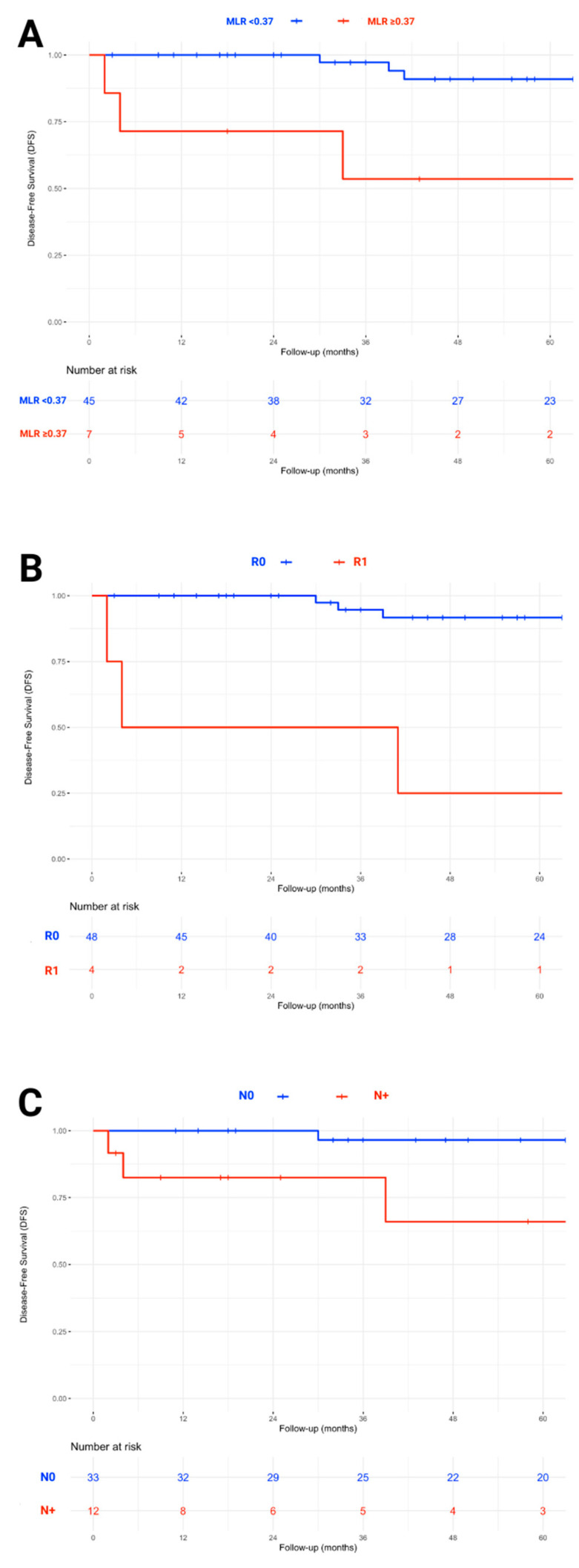
Kaplan–Meier curves for disease-free survival (DFS) according to (**A**) monocyte-to-lymphocyte ratio (MLR), (**B**) surgical margins, and (**C**) lymph node status in medullary thyroid carcinoma. Elevated monocyte-to-lymphocyte ratio (MLR ≥ 0.37), positive surgical margins (R1), and histological lymph node metastases (N+) were identified as independent predictors of worse DFS.

The results of the univariate analyses for DFS are summarized in Table 2. An MLR ≥ 0.37 (hazard ratio [HR] = 8.95, 95% CI: 2.22–36.03, *p*-value = < 0.01), a SIRI ≥ 1.56 (HR = 13.32, 95% CI: 2.41–73.59, *p*-value = < 0.01), the presence of ETE (HR = 8.22, 95% CI: 1.82–37.07, *p*-value = < 0.01), angioinvasion (HR = 4.62, 95% CI: 1.19–18.03, *p*-value = 0.03), positive surgical margins (HR = 7.05, 95% CI: 1.67–29.65, *p*-value = < 0.01), and positive pN status (HR = 23.31, 95% CI: 2.84–191.08, *p*-value = < 0.01) were all significantly associated with poorer DFS. Conversely, postoperative CT normalization was associated with a higher DFS (HR = 0.08, 95% CI: 0.02–0.41, *p*-value = < 0.01). Cox regression for the RET variable was not performed due to the absence of recurrences in the RET-mutated group, which led to model convergence issues and made it impossible to estimate the hazard ratio. The first and last steps of the backward stepwise selection multivariate analysis are shown in Table 2. Since there was strong collinearity between MLR and SIRI, only MLR, which showed higher significance, was included in the multivariate analysis. MLR (HR = 9.73, 95% CI: 1.41–67.22, *p*-value = 0.01), surgical margins (HR = 10.78, 95% CI: 1.44–80.39, *p*-value = 0.02), and pN status (HR = 17.71, 95% CI: 1.82–172.07, *p*-value = 0.02) were the only covariates independently associated with DFS. The final multivariable model demonstrated adequate fit (AIC = 25.51). Kaplan–Meier curves for DFS according to significant and independent prognostic factors are shown in Figure 1.

## 4. Discussion

Our study, which included 52 patients with MTC who underwent surgery with curative intent, demonstrated that MLR, surgical margins, and pN status are independently associated with DFS.

MTC, arising from thyroid C cells, is rare, accounting for 5% of thyroid cancers [1], and differs from more common papillary and follicular thyroid carcinomas not only in histology and origin but also in management and prognosis [2]. In our cohort, the overall 5-year DFS was 86%, aligning with current literature that reports a 5-year DFS ranging from 70% to 95% [18].

MTC tends to spread to the regional neck nodes, representing a negative prognostic factor. Our study confirmed this, finding an independent correlation between N status and DFS, with an HR of 17.71. However, these node metastases are often microscopic and not clinically detectable at diagnosis, leading to regional recurrences even years after surgery [2]. For this reason, there has been a long-standing debate over whether and when to proceed with END and, if so, the extent of such surgery [5,6]. One of the most studied predictive factors in this regard has been CT, a hormone produced by both the C cells from which MTC originates and the tumor itself. Considered as a marker of disease extent, achieving postoperative undetectable CT levels is a surgical goal, indicating the greatest possible radicality, which is associated with a better prognosis [4,19]. Conversely, an increase in CT levels during follow-up is highly suggestive of disease recurrence, often due to locoregional or distant recurrence.

Several studies have linked preoperative CT levels to nodal involvement, proposing different surgical management strategies based on preoperative CT. Following the retrospective study by Machens et al. [4], which included 224 patients, the European Society for Medical Oncology (ESMO) suggests ipsilateral END for calcitonin levels between 50 and 200 pg/mL and bilateral LND for calcitonin levels greater than 200 pg/mL. Juez et al. [20], in their retrospective multicenter study enrolling 244 patients, suggest a different approach based on both the hereditary or sporadic nature of the tumor and the preoperative CT levels, being more aggressive if the tumor is associated with a genetic alteration.

However, demonstrating an association between preoperative CT levels and disease extent differs from proving that increasing the extent of END improves outcomes. Other factors come into play, including the frequency of recurrence and the success rate of salvage treatment. A recent retrospective study by Spanheimer et al. [6] included 86 patients with clinically negative neck involvement and CT levels exceeding 200 pg/mL who underwent either END or not. The overall incidence of recurrences, disease-specific survival, and overall survival (OS) did not differ between the two groups, emphasizing that even if high CT levels correlate with greater disease extent, more aggressive surgery does not necessarily improve survival outcomes. Therefore, identifying other prognostic factors to select high-risk patients who could benefit from more aggressive elective treatment, sparing the others from overtreatment, seems essential.

The importance of the immune system in the progression of oncological diseases is now widely demonstrated. Indeed, several new drugs are capable of controlling malignant tumors by acting on specific pathways in the tumor–immunity interaction [21]. Moreover, the patient’s immunological status, assessed using simple blood markers, has shown considerable predictive value regarding survival, recurrence, and response to treatments in various types of tumors, including colorectal [22], breast [14], esophageal [23], and lung cancer [15]. These markers have also been studied in differentiated thyroid tumors, where they do not seem to have significant importance, perhaps due to the low aggressiveness and immunogenicity of these histological entities [24,25].

In our study, five immunological markers were calculated to assess their relationship with disease recurrence. MLR and SIRI were found to be significantly correlated with DFS. However, given their strong collinearity, only MLR was included in the multivariate analysis (as it was more statistically significant), which demonstrated an independent correlation between MLR and DFS. Specifically, elevated MLR values greater than 0.37 are an independent negative prognostic factor for disease recurrence.

Jiang et al. [26] also attempted to explore the role of MLR in predicting DFS in patients with MTC but did not find a significant correlation. However, the small sample size and the very short follow-up period may have limited the reliability of their findings. Their median follow-up was considerably shorter than in our cohort (75 months), potentially underestimating late recurrences typical of MTC. Importantly, they defined recurrence using biochemical parameters rather than structural confirmation, which may partly explain discrepancies with our findings. Li et al. [27], in their retrospective cohort study of sixty-eight patients, demonstrated a correlation between various immunological markers and postoperative CT progression, defined as a CT level increase to 150 pg/mL or a doubling time of less than 12 months. Although this study supports the hypothesis that these markers might be potential biomarkers of MTC, no data on confirmed recurrence or survival outcomes were presented. Conversely, while ours is the first study to demonstrate a potential link between MLR and DFS in MTC, the oncological significance of this marker has already been established in other tumor types, including those in the head and neck region. Although previous studies in MTC are limited and often exploratory in nature, the biological plausibility of MLR as a prognostic marker is strongly supported by extensive evidence in other solid tumors and head and neck malignancies. Therefore, our investigation should not be interpreted as isolated from prior research, but rather as an extension of a well-established oncologic inflammatory paradigm to a rare endocrine malignancy.

In the cohort of patient with oropharyngeal, hypopharyngeal, and laryngeal cancers by Kano et al. [13], a lymphocyte-to-monocyte ratio (the mathematically inverse of MLR) higher than 3.22 was an independent protective prognostic factor for OS, with an HR of 0.438. Furthermore, Mikoshiba et al. [12] demonstrated that the lymphocyte-to-monocyte ratio was statistically correlated with OS and DFS in parotid gland carcinoma. A possible explanation for these results may be that tumor-associated macrophages play a role in secreting pro-inflammatory cytokines, such as specific interleukins, tumor necrosis factor, and transforming growth factor-β, which promote tumor-associated angiogenesis, invasion, and migration [28,29]. At the same time, these same factors are responsible for suppressing anti-tumor immune processes [30]. In this context, MLR might reflect the systemic balance between host immune status and tumor malignancy; a higher monocyte representation compared to lymphocytes (higher MLR) indicates that the tumor is less controlled by the immune system [8].

It should also be noted that MLR may be influenced by patients’ comorbidities, such as chronic inflammatory conditions [31], autoimmune diseases [32], cardiovascular disorders [33], or infections [34]. These comorbidities can alter monocyte or lymphocyte counts, potentially confounding the interpretation of MLR as a tumor-related biomarker. In our series, although comorbidity data were collected, the relatively small number of patients presenting with such conditions did not allow for robust statistical inference on their impact on MLR.

The conclusions of this study are subject to several limitations. Firstly, the retrospective nature of this study is inherently at risk of selection and recall bias. To reduce potential confounding effects, major clinicopathological variables were included in multivariable modeling. Nevertheless, residual confounding inherent to retrospective designs cannot be entirely excluded. Additionally, the small sample size poses several statistical issues. One consequence is the very wide 95% CI observed, indicating that the magnitude of the risk associated with the analyzed variables is not easily describable, even if statistically significant. Furthermore, the small cohort size might have underestimated the importance of some factors that therefore did not result in statistical significance, even when recognized in the literature as important, such as primary tumor size, age, and male gender. Moreover, the low event rate further compounds these issues by increasing the risk of model overfitting, whereby the predictive performance of the statistical model may be artificially inflated within the study dataset but fail to generalize to other populations. Therefore, these findings should be considered hypothesis-generating rather than definitive. Finally, it is well known that the RET mutation plays a crucial role in the pathogenesis and progression of MTC, particularly in hereditary cases. Identification of RET mutations has important implications for prognosis, surgical planning, and genetic counseling of patients and their families. Unfortunately, in our cohort, RET status was available for only a subset of patients, which limited the possibility of including this variable in our survival analysis and fully assessing its prognostic value.

A consensus on MTC management, especially in cN0 patients, is still lacking [35]. On one hand, preoperative CT levels seem to be a good marker for deciding when and how much to extend END; on the other hand, there is still no evidence that this approach results in improved outcomes. In this context, MLR, an inexpensive marker, easy to calculate from the complete blood count that all patients routinely undergo preoperatively, may be added to the pool of tools useful for identifying MTC patients at higher risk who could benefit from more aggressive treatments and stricter follow-up. However, further larger, multi-institutional prospective studies are strongly required to validate the findings of this study and to investigate the effect of adjusted treatment protocols that consider MLR as a prognostic factor for patients with MTC.

Additionally, future studies could also investigate variations between preoperative and postoperative MLR, evaluating whether they correlate with recurrence risk or treatment response. Such a dynamic assessment might provide additional follow-up tools and prognostic information beyond baseline measurements, potentially enabling earlier detection of unfavorable disease courses. Future research should incorporate predefined subgroup or adjusted analyses accounting for active infections, chronic inflammatory conditions, autoimmune diseases, and cardiovascular disorders, as these factors may independently influence circulating monocyte and lymphocyte counts. Larger prospectively collected cohorts would allow more robust evaluation of these potential confounders and of prognostic models integrating MLR with established biomarkers (e.g., CT, RET).

## 5. Conclusions

This study revealed that a MLR ≥ 0.37, the presence of pathological nodes at final histology, and positive surgical margins are useful predictors of DFS in patients with MTC who have undergone curative treatment.

MLR is an inexpensive immunological marker that can be easily obtained from routine preoperative blood tests. Considering that patients with a higher MLR have a greater risk of developing recurrence after treatment, they may benefit from more aggressive elective treatments and/or more intensive follow-up.

Larger multicenter prospective studies are necessary to confirm these findings and MLR’s role in identifying patients at high risk of recurrence.

## Figures and Tables

**Table 1:** Characteristics of the included patients and corresponding 3- and 5-year disease-free survival (DFS) rates, with statistical significance assessed using the log-rank test. Abbreviations: DM, Diabetes Mellitus; HTN, Hypertension; US, Ultrasound; preCT, Preoperative Calcitonin; MLR, Monocyte-to-Lymphocyte Ratio; NLR, Neutrophil-to-Lymphocyte Ratio; PLR, Platelet-to-Lymphocyte Ratio; SII, Systemic Inflammatory Index; SIRI, Systemic Inflammatory Response Index; Hdim, Histological Dimension; ETE, Extrathyroidal Extension; R0/R1, Resection status (Complete/Incomplete); CND, Central Neck Dissection; LND, Lateral Neck Dissection; RET, Rearranged during Transfection (RET) Mutation; CT, Calcitonin.

Variable				3-Year DFS	5-Year DFS	*p*-Value
Overall			N = 52	0.91	0.86	
Age			52 (100%)			
		Mean (range)	55.0 (31–75)			
		≤56	27 (52%)	0.92	0.87	0.432
		>56	25 (48%)	0.91	0.85	
Gender			52 (100%)			
		Female	38 (73%)	0.93	0.85	0.814
		Male	14 (27%)	0.85	0.85	
Smoking			52 (100%)			
		No	43 (83%)	0.92	0.89	0.379
		Yes	9 (17%)	0.86	0.71	
DM			52 (100%)			
		No	45 (87%)	0.92	0.86	0.383
		Yes	7 (13%)	0.86	0.86	
HTN			52 (100%)			
		Yes	35 (67%)	0.90	0.86	0.960
		No	17 (33%)	0.94	0.86	
US Findings			37 (71%)			
	Mean Dimension (range, [mm])		14.1 (3.0–62.0)			
	Microcalcification (Yes/No)		14 (%)/23 (%)			
	Echogenicity (Hypo/Hyper/Mixed)		27 (%)/2 (%)/8 (%)			
Clinical TNM classification			48 (92%)			
	cT (1A/1B/2/3A)		23 (48%)/14 (29%)/7 (15%)/4 (8%)			
	cN (0/1A/1B)		43 (90%)/1 (2%)/4 (8%)			
preCT			50 (96%)			
		Mean (range, [pg/mL])	570.9 (1.4–4983.5)			
		<181.0 pg/mL	28 (56%)	0.94	0.94	0.031
		≥181.0 pg/mL	22 (44%)	0.86	0.77	
Preop Blood Immune Markers			52 (100%)			
	NLR					
		Mean (range)	2.31 (0.56–4.90)			
		<2.46	30 (58%)	0.97	0.87	0.076
		≥2.46	22 (42%)	0.84	0.84	
	MLR					
		Mean (range)	0.28 (0.09–0.86)			
		<0.37	45 (87%)	0.97	0.91	<0.001
		≥0.37	7 (13%)	0.54	0.54	
	PLR					
		Mean (range)	135.71 (38.57–297.14)			
		<193.12	47 (90%)	0.93	0.87	0.078
		≥193.12	5 (10%)	0.75	0.75	
	SII					
		Mean (range)	558.56 (150.43–1274.00)			
		<418.70	16 (31%)	0.93	0.85	0.497
		≥418.70	36 (69%)	0.90	0.86	
	SIRI					
		Mean (range)	1.17 (0.33–2.94)			
		<1.56	38 (73%)	1.00	0.93	<0.001
		≥1.56	14 (27%)	0.64	0.64	
Surgery			52 (100%)			
	T Surgery (TT/HT)		49 (94%)/3 (6%)			
	N Surgery (CND/LND/None)		42 (81%)/6 (12%)/10 (19%)			
Histological Findings			52 (100%)			
	Hdim (mm)					
		Mean (range)	13.7 (3.0–62.0)			
		<11	32 (62%)	1.00	0.90	0.042
		≥11	20 (38%)	0.79	0.79	
	ETE					
		No	48 (92%)	0.93	0.90	< 0.01
		Yes	4 (8%)	0.75	0.50	
	Angioinvasion					
		No	41 (79%)	0.95	0.95	0.017
		Yes	11 (21%)	0.78	0.52	
	Surgical Margins					
		R0	48 (92%)	0.95	0.92	<0.001
		R1	4 (8%)	0.50	0.25	
	Multifocality					
		No	47 (90%)	0.90	0.85	0.412
		Yes	5 (10%)	1.00	1.00	
CND Lymph Nodes			42 (81%)			
	Mean Retrieved (range)		7.1 (0.0–27.0)			
	Mean Positive (range)		0.8 (0.0–11.0)			
LND Lymph Nodes			6 (12%)			
	Mean Retrieved (range)		40.5 (0.0–78.0)			
	Mean Positive (range)		8.3 (0.0–26.0)			
Pathological TNM Classification			52 (100%)			
	pT (1A/1B/2/3A/3B/4)		31 (60%)/11 (21%)/4 (7%)/3 (6%)/3 (6%)			
		T1/2	46 (88%)	0.92	0.89	0.100
		T3/4	6 (12%)	0.83	0.67	
	pN (0/1A/1B)		40 (77%)/8 (15%)/4 (8%)			
		N0	33 (73%)	1.00	1.00	<0.001
		N+	12 (27%)	0.74	0.53	
RET mutation			39 (75%)			
		No	33 (85%)	0.93	0.85	0.422
		Yes	6 (15%)	1.00	1.00	
CT Normalization			49 (94%)			
		Yes	39 (80%)	0.97	0.97	<0.001
		No	10 (20%)	0.79	0.56	

**Table 2:** Univariable and multivariable Cox proportional hazards regression analyses for disease-free survival (DFS). The first and final stages of the backward stepwise selection process are shown. The Akaike Information Criterion (AIC) is reported for both stages to indicate model quality; lower AIC values reflect better model performance. Abbreviations: HR, Hazard Ratio; 95% CI, 95% Confidence Interval; preCT, Preoperative Calcitonin; NLR, Neutrophil-to-Lymphocyte Ratio; MLR, Monocyte-to-Lymphocyte Ratio; PLR, Platelet-to-Lymphocyte Ratio; SII, Systemic Inflammatory Index; SIRI, Systemic Inflammatory Response Index; Hdim, Histological Dimension; ETE, Extrathyroidal Extension; R0/R1, Resection Status (Complete/Incomplete); CT, Calcitonin.

Variable	Univariable Analysis			Multivariable Analysis					
	HR	95% CI	*p*-Value	First Stage (Partial AIC = 38.43)			Last Stage (Partial AIC = 25.51)		
				HR	95% CI	*p*-Value	HR	95% CI	*p*-Value
Age									
≤56	-	-							
>56	1.69	0.45–6.33	0.44						
Sex									
Female	-	-							
Male	1.18	0.29–4.76	0.81						
Smoking									
No	-	-							
Yes	2.05	0.40–10.40	0.39						
DM									
No	-	-							
Yes	2.03	0.40–10.30	0.39						
HTN									
Yes	-	-							
No	1.04	0.24–4.44	0.96						
preCT (pg/mL)									
<181.0	-	-		-	-				
≥181.0	7.23	0.89–58.59	0.06	0.58	0.01–28.54	0.78			
NLR									
<2.46	-	-							
≥2.46	3.28	0.82–13.18	0.19						
MLR									
<0.37	-	-		-	-		-	-	
≥0.37	8.95	2.22–36.03	<0.01	9.38	0.57–153.90	0.12	9.73	1.41–67.22	0.01
PLR									
<193.12	-	-							
≥193.12	3.85	0.77–19.31	0.10						
SII									
<418.70	-	-							
≥418.70	1.72	0.35–8.31	0.50						
SIRI									
<1.56	-	-							
≥1.56	13.32	2.41–73.59	<0.01						
Hdim (mm)									
<11	-	-		-	-				
≥11	4.54	0.92–22.52	0.06	1.65	0.17–15.70	0.66			
ETE									
No	-	-		-	-				
Yes	8.22	1.82–37.07	<0.01	0.69	0.06–8.08	0.77			
Angioinvasion									
No	-	-		-	-				
Yes	4.62	1.19–18.03	0.03	1.39	0.11–18.52	0.80			
Surgical Margins									
R0	-	-		-	-		-	-	
R1	7.05	1.67–29.65	<0.01	12.85	0.78–211.72	0.07	10.78	1.44–80.39	0.02
Multifocality									
Yes	-	-							
No	1.63	0.19–13.59	0.65						
pT Classification									
T1/2	-	-							
T3/4	3.13	0.75–12.98	0.12						
pN Classification									
N0	-	-		-	-		-	-	
N+	23.31	2.84–191.08	<0.01	18.95	0.20–1780.45	0.20	17.71	1.82–172.07	0.02
CT Normalization									
No	-	-		-	-				
Yes	0.08	0.02–0.41	<0.01	0.92	0.05–17.10	0.96			

## Data Availability

The original contributions presented in this study are included in the article. Further inquiries can be directed to the corresponding author.
